# Supplementary material for: In vivo generation of a mature and functional artificial skeletal muscle
Source: EMBO Mol Med. 2015 Feb 25;7(4):411–22. doi: 10.15252/emmm.201404062 (PMC4403043; doi:10.15252/emmm.201404062)
Supplement: Supplementary file 1 — Supplementary Information [file emmm0007-0411-sd1.docx]

**Supplementary Information**

**Supplementary Fig. 1.** Mabs differentiation time course in 2D standard culture plastic versus 3D PF environment.

**Supplementary Fig. 2.** PlGF transduction does not influence myogenic differentiation while promotes blood vessel recruitment.

**Supplementary Fig. 3.** Scheme of supernumerary generation artificial muscle.

**Supplementary Fig. 4.** Quantitative PCR analysis of RNA extracts from normal tibialis anterior (TA) muscles and their relative artificial muscles revealing comparable expression for muscle specific genes.

**Supplementary Fig. 5.** Immunofluorescence analyses on artificial muscle section revealing innervation at early and mature stage of implant development.

**Supplementary Fig. 6.** Analysis of *in vitro* vs *in vivo* LacZ positive mMabs as percentage of total labelled nuclei.

**Supplementary Fig. 7.** MyHC and GFP immunofluorescence on sections from PF embedded mMabs implanted into ubiquitous GFP expressing mouse background.

**Supplementary Fig. 8.** Histological analyses on regenerating artificial muscle section 3 days and 2 weeks after Cardiotoxin (CTX) treatment.

**Supplementary Fig. 9.** Immunofluorescence analysis on muscle satellite cells isolated from damaged artificial and TA muscles.

**Supplementary Fig. 10.** Hypertrophic and atrophic stimuli influence size and CSA of artificial and TA muscles.

**Supplementary Fig. 11.** *In vitro* and *in vivo* muscle differentiation of human myogenic precursor (hMabs) in PF hydrogels.

**Supplementary Fig. 12.** Dimension of dislodged TA tissue versus normal TA.

**Supplementary Fig. 13.** Diagram elucidating TA ablation experimental procedures.

**Supplementary Fig. 14**. Immunoflorescence on cross section from acellular PF and PF embedded Mabs grafted in an ablated TA injury, revealing artifical muscle formation after 8weeks.

**Supplementary Tab. 1.** Table showing number of satellite cell clones (either LacZ positive and/or negative) derived from artificial and TA muscles.

**Supplementary Tab. 2.** Table summarizing *in vivo* experimental groups.

**Supplementary Movie 1.** Mature contracting myotube movie revealing PF hydrogel influence on promoting m-Mabs originated muscle fibres maturation 5 days after cell encapsulation into the scaffold.

**Supplementary Movie 2.** 15 days m-Mabs culture encapsulated into 8mg/ml PF mould showing mature myofibres presenting remarkable contracting activity passing to the hole mould.

**Supplementary Movie 3.** Enlarged view of movies S3, revealing thicker and knotty network of mature and contracting m-Mabs derived myofibres.

**Supplementary Movie 4.** Surgical procedure showing TA dislodging and PF embedded Mabs-PlGF polymerization replacing ablated muscle.

**Supplementary Movie 5**. Limited walking activity of TA ablated mouse at one week after surgical operation, due to no flexing foot.

**
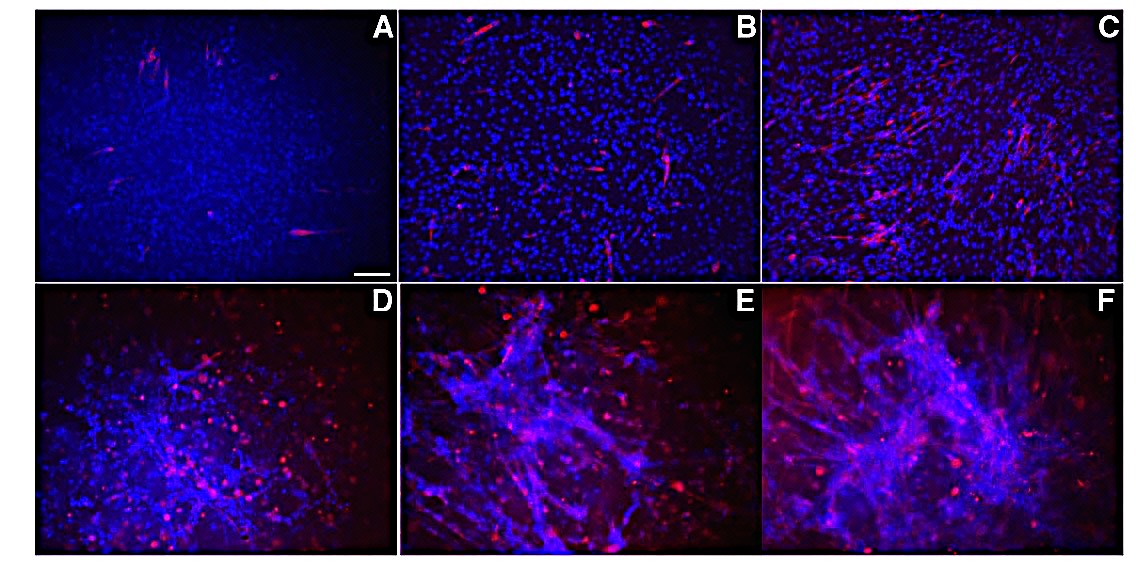
**

**Supplementary Fig. 1. Time course of Mabs differentiation on standard plastic culture and PF.** Immunofluorescence against MyHC (red) on differentiating Mabs grown on plastic (**A-C**) and embedded into PF (**D-F**). (**A, D**) 24 hours (**B, E**) 48 hours and (**C, F**) 96 hours after seeding. Nuclei are counterstained by DAPI (blue). Scale bar 50μm.

**
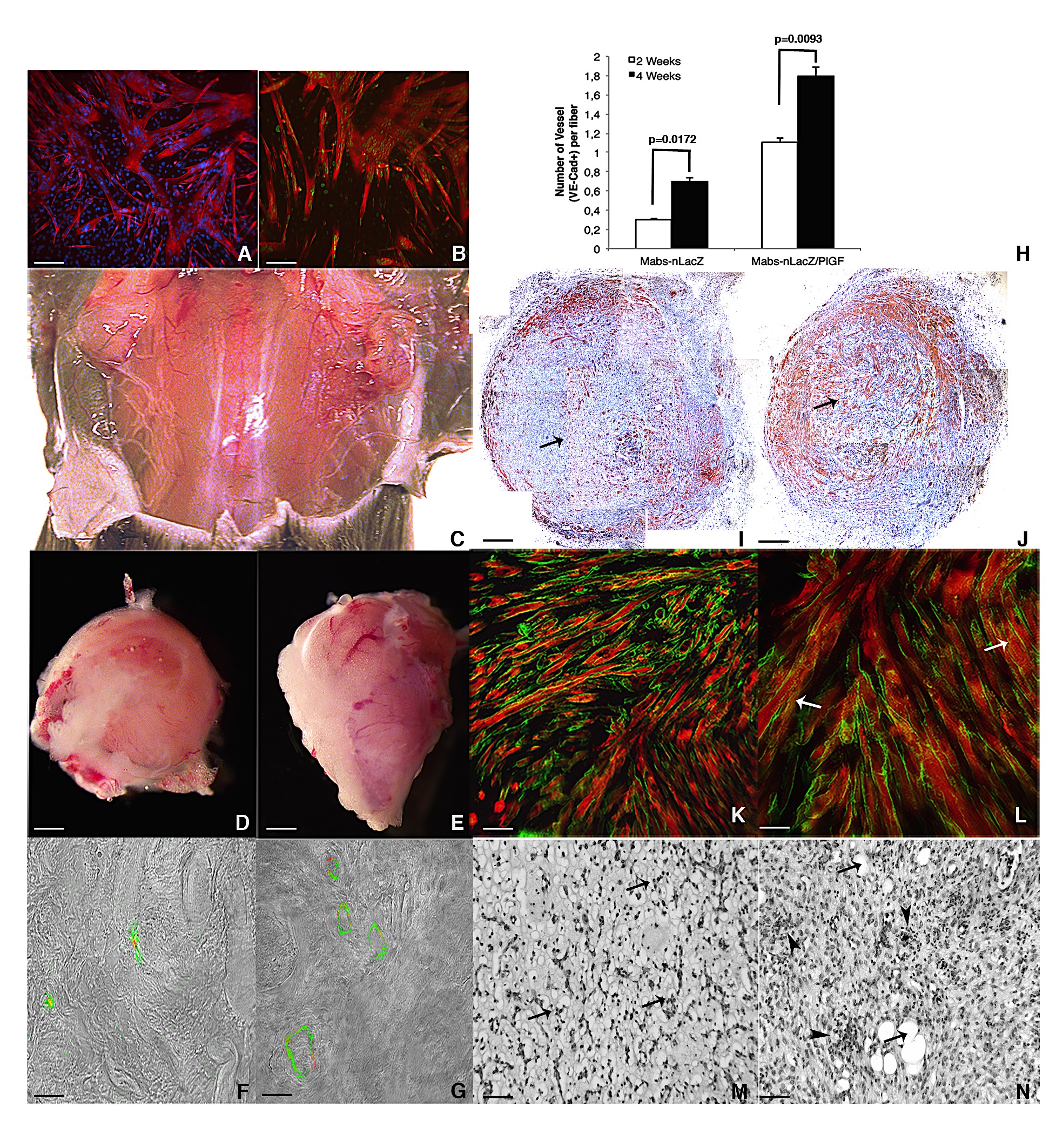
**

**Supplementary Fig. 2**. **PlGF transduction does not influence myogenic differentiation while promotes blood vessel recruitment.** (**A, B**) Immunofluorescence staining for MyHC (red) and LacZ (green), counterstained with DAPI­ labelling nuclei (blue) for wild type mouse Mabs (mMabs) (A) and mMabs-nLacZ expressing PlGF (m-Mabs-nLacZ/PlGF) (B), revealing that mMabs myogenic differentiation is not affected by lentiviral transduction and/or by PlGF expression. (**C-E)** Macroscopic comparison between *in vivo* dorsal subcutaneous mMabs-PF implant loaded with: 1,5x10^6^ mMabs-nLacZ/PlGF on the right and 1,5x10^6^ mMabs-nLacZ on the left, revealing increased blood vessel recruitment when PlGF is expressed. (**F-G)** Immunofluorescence analysis for Smooth Muscle Actin (SMA) (green) and VE-cad (red on mMabs-PF dorsal subcutaneous implant (4 weeks) sections expressing (G) or not (F) PlGF, showing increased blood vessels number under PlGF influence. (**H)** Capillary/muscle fibre ratio calculated at different time points (2 and 4 weeks after implantation) by scoring VE-cad-positive vessels, highlighting the increased number of blood vessel in the mMabs-PF implants expressing PlGF. (Group of n=5 of mMabs-PF expressing or not expressing PlGF has been analysed, values are presented as means ± standard error and statistical significance has been tested using Student’s t-test, *P* < 0.05 was considered significant (*). **(I-J)** MyHC (red) immunohistochemistry on *in vivo* subcutaneous implant section 2 weeks after transplantation, revealing differentiated muscle fibres in the centre (arrows) of PF embedded mMabs-nLacZ/PlGF implant (J) and reduced differentiation in the centre (arrows) of PF embedded mMabs implant. Haematoxylin labelled nuclei (blue) (I). (**K-L)** Laminin (green) and MyHC (red) immunofluorescence analysis revealing time dependent formation of large and mature myofibres as indicated by the cross-striations (arrows) at 4 weeks (L) not yet visible at 2 weeks (K). **(M-N)** Haematoxylin stained implant sections from PF embedded mMabs-nLacZ/PlGF at 3 (M) and 7 days (N) after subcutaneous implantation, showing PF encapsulated cells at 3 days (arrows in M) while at 7 days the PF has been mainly resorbed, the cells are aggregated in cluster lacking the hydrogel (arrowhead in N) minimal residual of PF has been found (arrows in N). Scale bar values: (A, B) 50μm, (C) 300μm, (D, E) 100μm, (F, G) 10μm, (I, J) 100μm, (K, L) 20μm, (M, N) 50μm.


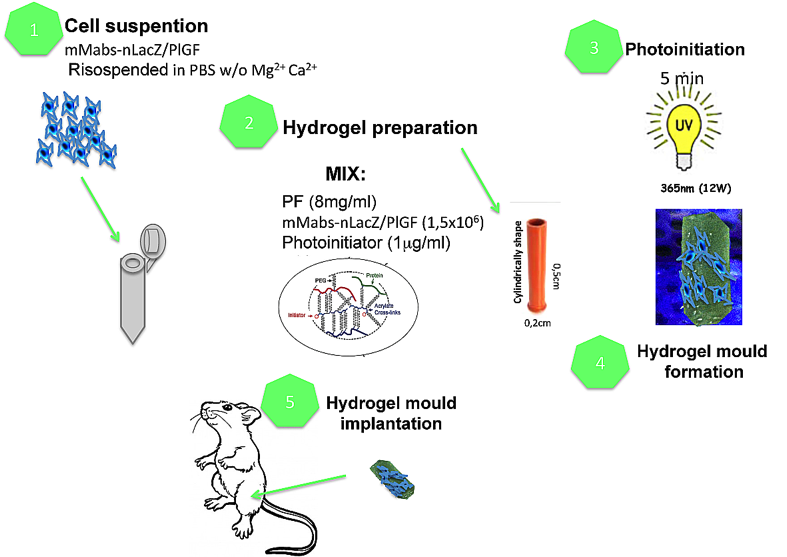


**Supplementary Fig. 3. Diagram depicting experimental procedures for supernumerary artificial muscle generation.**


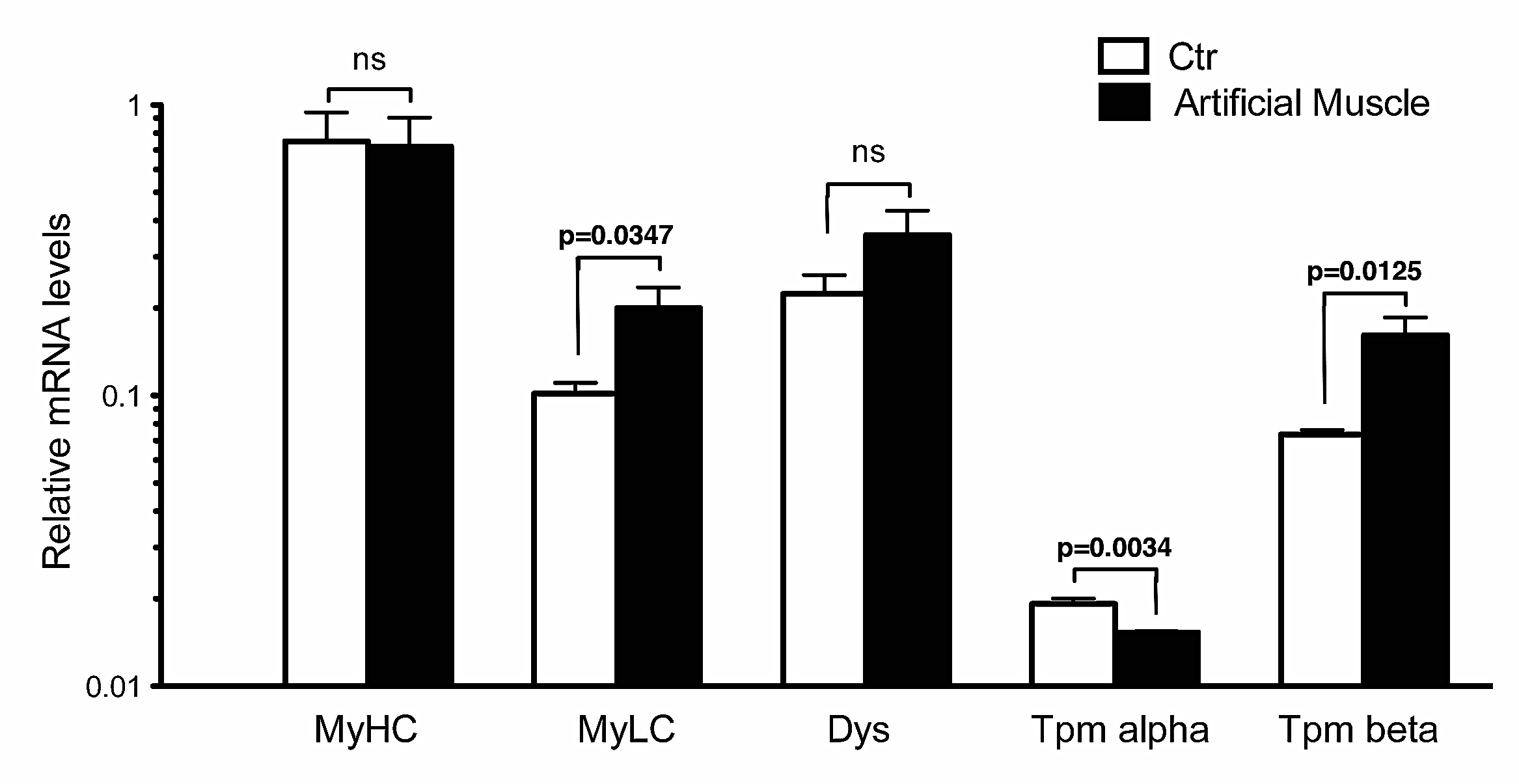


**Supplementary Fig. 4**. **Quantitative PCR analysis of RNA extracts from normal tibialis anterior (TA) muscles (white columns, n=5) and their relative artificial muscles (black columns, n=5) revealing comparable expression for the following genes: Myosin Heavy Chain (MyHC), Myosin Light Chain (MyLC), Dystrophin (Dys), Tropomyosin alpha (Tpm alpha) and beta (Tpm beta)**. Data are expressed as mRNA level relative to the house-keeping gene GAPDH. Values refer to a TA muscle of a WT mouse, which has been used as a positive control and calibrator (set as 1). Error bars represent means of three experiments ± standard error and statistical significance has been tested using Student’s t-test (*P* < 0.05 was considered significant); ns: not significant.

**
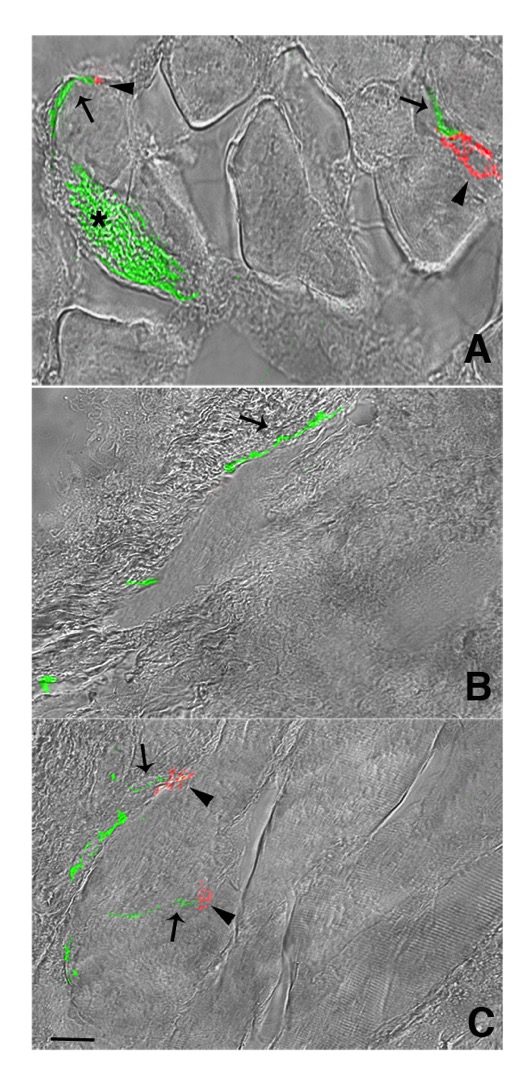
**

**Supplementary Fig. 5**. **Immunofluorescence analyses on artificial muscle section revealing innervation at early and mature stage of implant development showing neurofilament (green) and bungarotoxin (red) immunostaining mounted over phase contrast image.** (**A**) Mature host TA revealing axon (arrow) contacting neuro-muscular plaque (arrowhead), asterisk indicating chunk of nerve; (**B)** Early stage (4 weeks after implantation) artificial muscle showing only axon (arrow) flanking a myofibre; (**C**) Late stage (8 weeks after implantation) artificial muscle revealing complete innervation represented by axon (arrow) contacting neuro-muscular plaque (red) as shown in the control TA. Scale bar values: (A) 10μm, (B, C) 30μm.


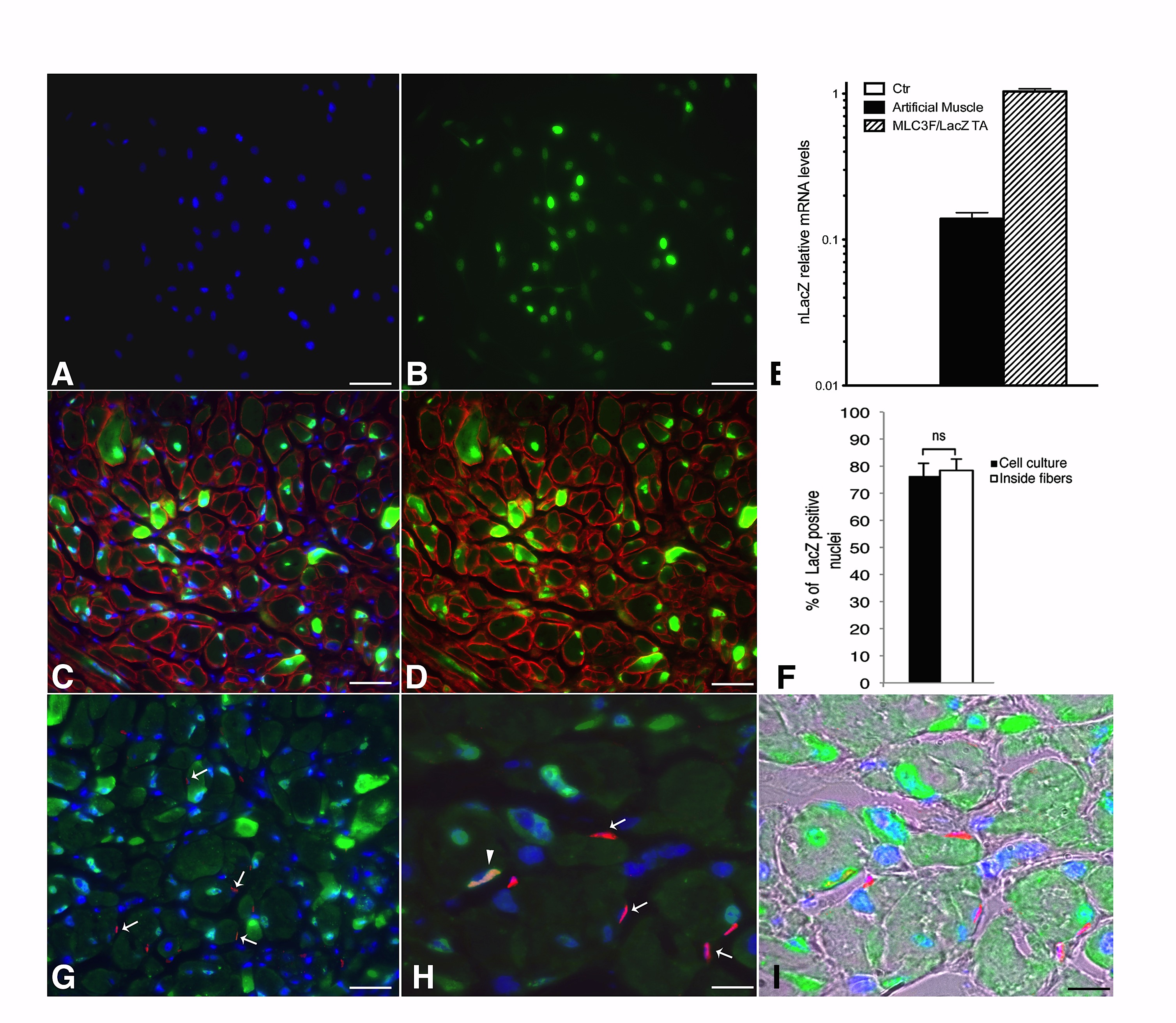


**Supplementary Fig. 6**. **Analysis of *in vitro* vs *in vivo* LacZ positive mMabs as percentage of total nuclei.** (**A, B**) mMabs-nLacz/PlGF standard cell culture counterstained with DAPI labelling nuclei (blue) (A) showing the percentage of positive LacZ (green) cells by anti-LacZ immunofluorescence (B). (**C, D**) Immunofluorescence on artificial muscle section (8 weeks after implantation) revealing dystrophin expression (red) at the fibre membrane, surrounding many LacZ positive nuclei (green) and negative DAPI counterstained nuclei (blue) in (C) localized mainly in the extracellular matrix. (**E)** Expression of nLacZ reporter gene of a not treated tibialis anterior muscle (TA) (white column, n=5) compared with artificial muscles (black column, n=5) and with the TA from a MLC/3F-nLacZ reporter mouse, where all skeletal muscle nuclei express LacZ, used here as positive control. (**F**) Graphic representing the percentage of LacZ positive nuclei *in vivo* scored from five 40X randomly selected fields of 3 different sections from each sample (n=5). Values are presented as means ± standard error and statistical significance has been tested using Student’s t-test (*P* < 0.05 was considered significant). (**G)** LacZ (green) and Pax7 (red) double immunofluorescence on artificial muscle section revealing stem cell niche constitution, the nuclei are DAPI (blue) counterstained. (**H)** (G) high magnification indicating the presence of satellite cells (arrows) being occasionally LacZ positive (arrowhead), nuclei are DAPI (blue) labelled. (**I)** immunostaining in (H) mounted over phase contrast image showing Pax7 (red) labelled satellite cells localized outside muscle fibres . Scale bar value: (A-D) 50μm;(G) 50μm; (H, I) 20μm.


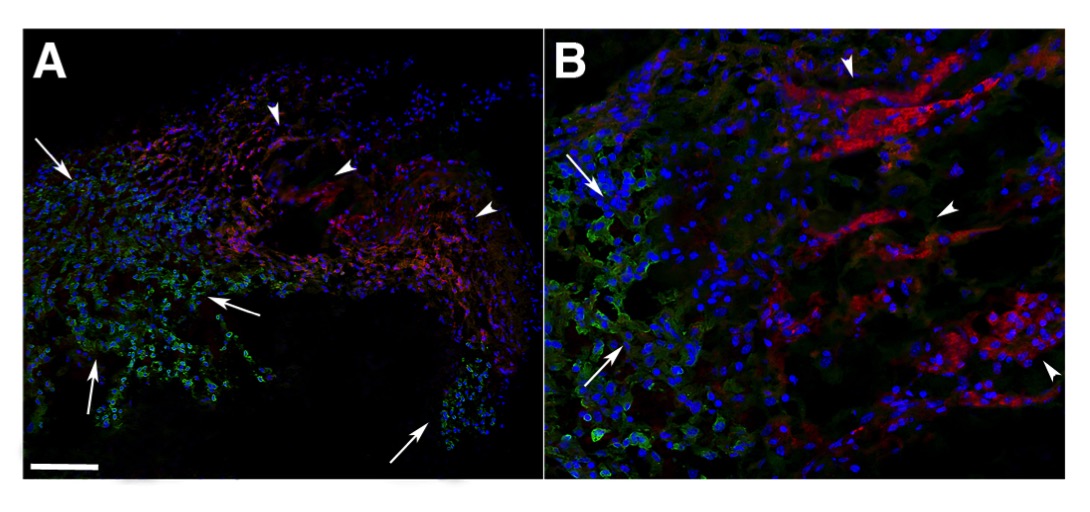


**Supplementary Fig. 7. Implantation of PF embedded Mabs/PlGF construct on GFP mouse host background.** (A) Immunofluorescence against GFP (green) and MyHC (red) on sections from ubiquitous GFP expressing mice hosting PF embedded Mabs/PlGF showing host cells GFP positive (arrows) surrounding implanted cells differentiating into muscular tissue (arrowheads) after two weeks. (B) Enlarged view revealing absence of GFP positivity (arrows) in the differentiating implanted structure (arrowheads) derived from PF embedded Mabs/PlGF. Scale bar (A) 100μm, (B) 30μm.


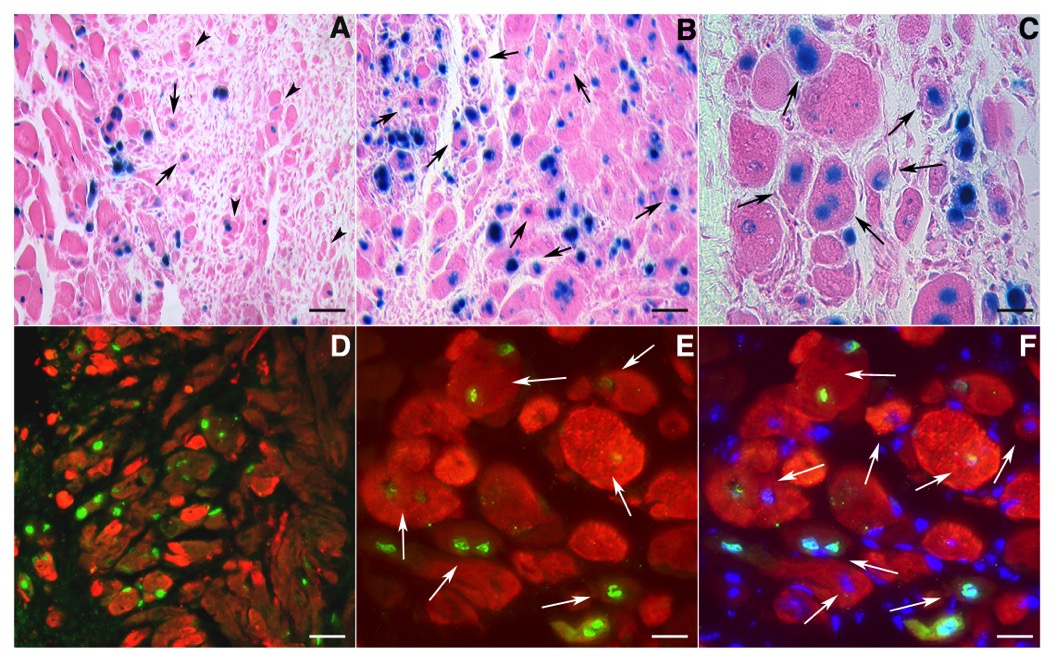


**Supplementary Fig. 8. Histological analyses on regenerating artificial muscle section 3 days (A) and 2 weeks (B-F) after Cardiotoxin (CTX) treatment.** (**A)** X-Gal staining (blue) on artificial muscle section revealing muscle degeneration (arrowhead) and regeneration process beginning (arrows) on Mabs-nLacZ originated artificial muscle 48 hours after CTX muscle crush. (**B)** LacZ histochemical staining (blue) on artificial muscle section revealing regeneration process on going on mMabs-nLacZ originated artificial muscle 2 weeks after CTX muscle crush. (**C)** (B) Enlarged view showing numerous lacZ positive (blue) centre nucleated muscle fibres (arrows), representing regenerating artificial muscle myofibres. Histological sections (A-C) were counterstained by H&E. (**D)** Immunofluorescence on artificial muscle labelling in red Myosin Heavy Chain and in green LacZ showing mMabs-nLacZ derived regenerating artificial muscle 2 weeks after CTX injection. (**E, F)** (D) Enlarged view revealing several LacZ positive centro-nucleated regenerating muscle fibres (arrows), DAPI counterstaining labelling nuclei (blue) in (F). Scale bar value: (A, D) 50μm; (b) 30μm; (C, E, F) 10μm.


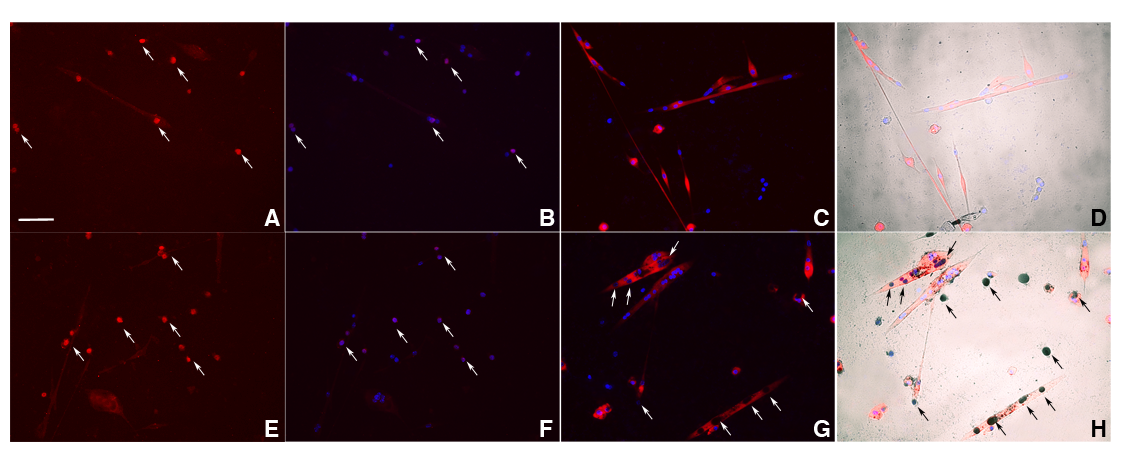


**Supplementary Fig. 9. Myogenic differentiation from artificial muscle isolated satellite cells.** Immunofluorescence against Pax7 and MyHC (red) on isolated satellite cells derived from underlying TA control (A-D) and artificial muscle (E-H). (A, B, E, F) Immunofluorescence on freshly isolated satellite cells revealing Pax7 expression (arrows). (B, F) nuclei were counterstained with DAPI (blue). (C, D, G, H) Immunofluorescence with anti MyHC antibody (red) on differentiating satellite cells; nuclei were counterstained by DAPI (blue); nLacZ positive nuclei (arrows) are easily detected both in mononucleated cells and in myotubes derived from artificial muscle (H) but not from the underlying TA (D). In D and H MyHC immunofluorescence was superimposed on X-Gal staining phase contrast images. Scale bar 50μm.


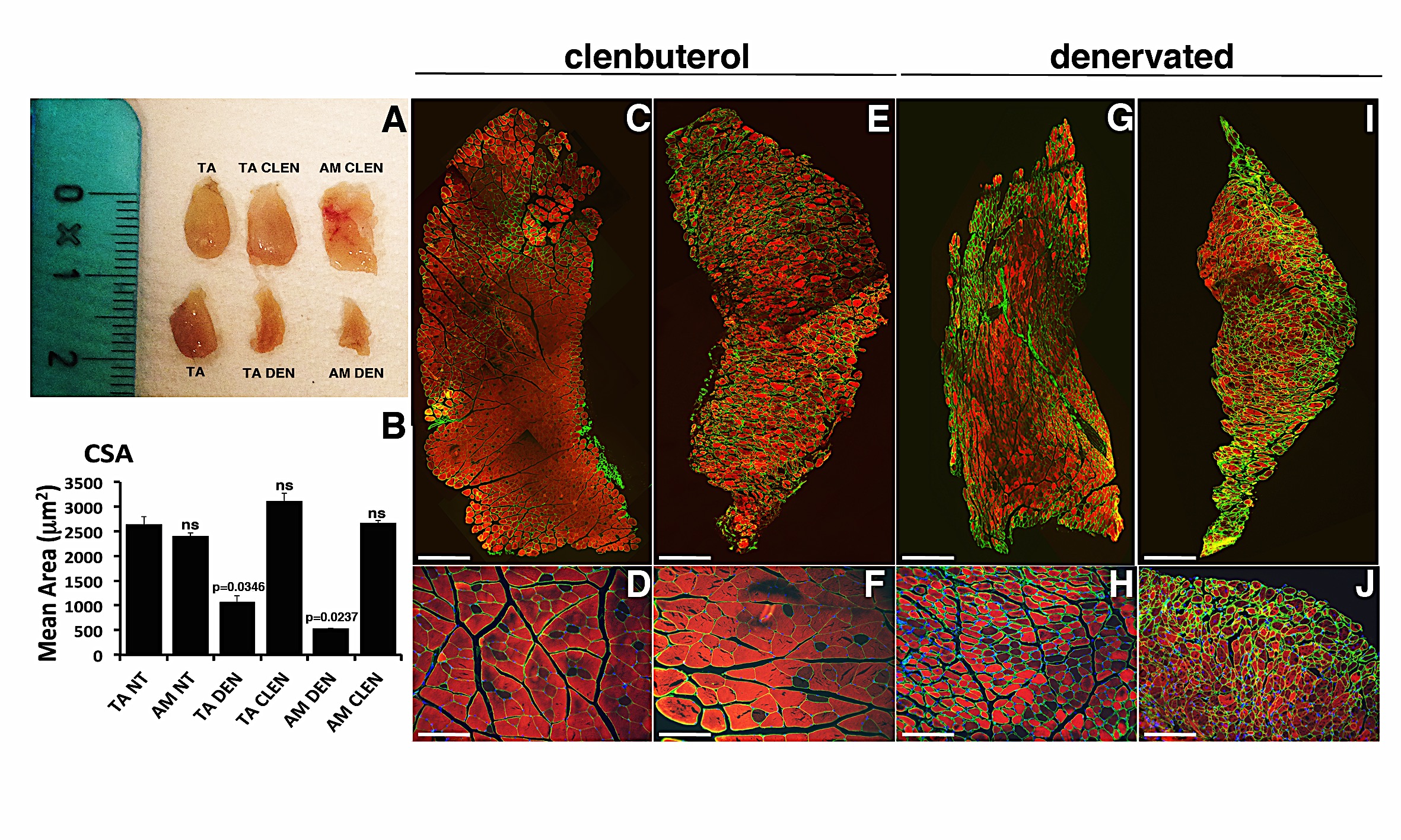


**Supplementary Fig. 10. Hyper and atrophy induction in artificial muscle.** (A) Gross morphology image showing the size of control TA and artificial muscle (am) subjected to hypertrophic (clenbuterol) and atrophic (denervation) stimuli. (B) CSA average analyzed on sections of different artificial and TA samples (group of n=4) treated for hypertrophy (clenbuterol) and atrophy (denervation); the values are expressed as means ± standard error and statistical significance has been tested using Student’s t-test, *P* < 0.05 was considered significant. (C-J) Representative immunofluorescence against MyHC (red) and Laminin (green) on TA (C, G) and artificial muscle (E, I) reconstructed sections revealing architecture organization and fiber size under hypertrophic (C, E) and atrophic (G, I) signals. (D-J) Higher magnification from (C-J) demonstrating muscle fiber size details under hypertrophic (D, F) and atrophic (H, J) stimuli, nuclei are labeled in blue by DAPI. Scale bars: (A-D) 500μm, (E-H) 50μm.


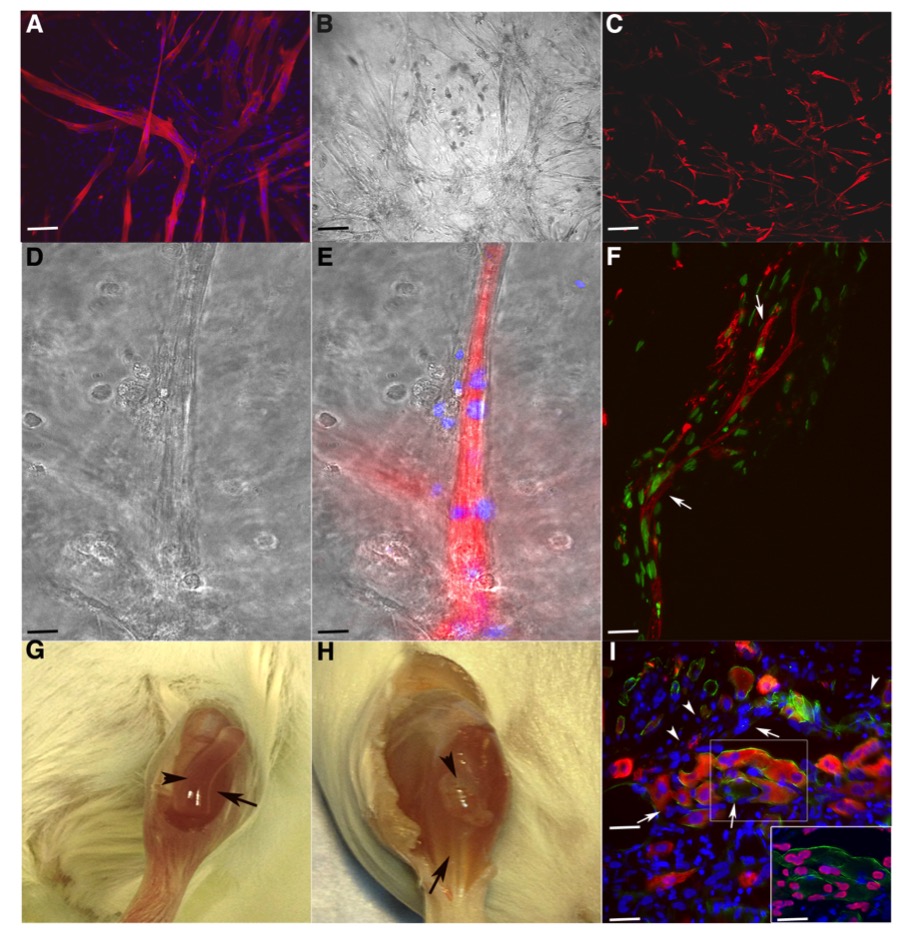


**Supplementary Fig. 11**.  ***In vitro* and *in vivo* muscle differentiation of human myogenic precursor (hMabs) in PF hydrogels. (A)** MyHC (red) immunofluorescence on hMabs standard cell culture 10 days after cell seeding showing myo-differentiation capability, DAPI counterstaining labeling nuclei (blue). **(B**) Phase contrast image of differentiated hMabs in PF hydrogels 5 days after cell seeding reveal a thick three-dimensional (3D) network of well formed myotubes. (**C**) Immunofluorescence labeling of MyHC (red) showing hMabs differentiated into myotubes in the PF hydrogels. (**D**) High magnification phase contrast image focusing on a human derived myofibre developing in the PF. (**E**) Immunofluorescence against MyHC (red) superimposed on a phase contrast image (D) revealing multicellular myogenic differentiation in the PF hydrogels; nuclei labeled with DAPI (blue) are located at the periphery of the myofibre. (**F**) *In vitro* construct of hMabs in PF labeled by immunofluorescence for MyHC (red) and Lamin A/C (green), showing well differentiated myofibres (labeled in red), with cross striations (arrows) and human positive nuclei (labeled in green) after 5 days (**G-H**) *In vivo* construct of hMabs in PF (arrowhead) implanted subcutaneously on the surface of a mouse TA (arrows) at the time of grafting (G) and after 30 days (H), revealing muscle like tissue formation (n=5). (**I**) Immunofluorescence analysis with antibodies against MyHC (red) and Laminin (green) on the muscle-like tissue sections revealing a *quasi* complete organization of the human artificial muscle in the host mouse. Shown is the myosin (red) expressing cytoplasm surrounded by laminin (green), with human nuclei (arrows) labeled by DAPI (blue) inside the differentiated muscle fibres and mouse nuclei (arrowhead), with the characteristic stippled patterns surrounding the human derived implant. Inset shows an enlarger region, immunolabeled for Lamin A/C (red) of the human-derived hMabs nuclei surrounded by Laminin (green), and DAPI-labeled host derived nuclei (blue). Scale bar values: (A, B) 50μm, (C) 100μm, (D, E) 10μm, (F, I, inset) 20μm.


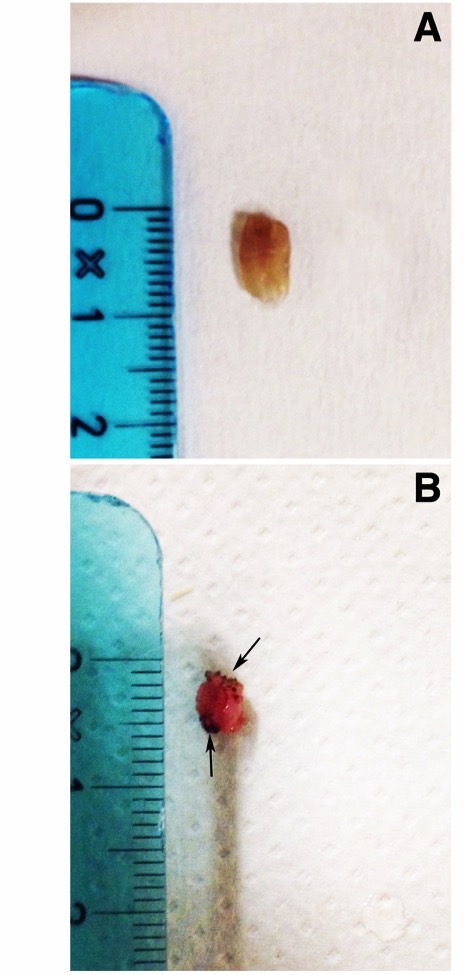


**Supplementary Fig. 12. Dimension of dislodged TA in comparison with a normal TA.** Photographs showing the dimensions of the normal TA (A) comparing with the ablated TA tissue (B) presenting black burning (arrows) due to the surgical procedure and revealing size of approximately 80-90% of normal TA.


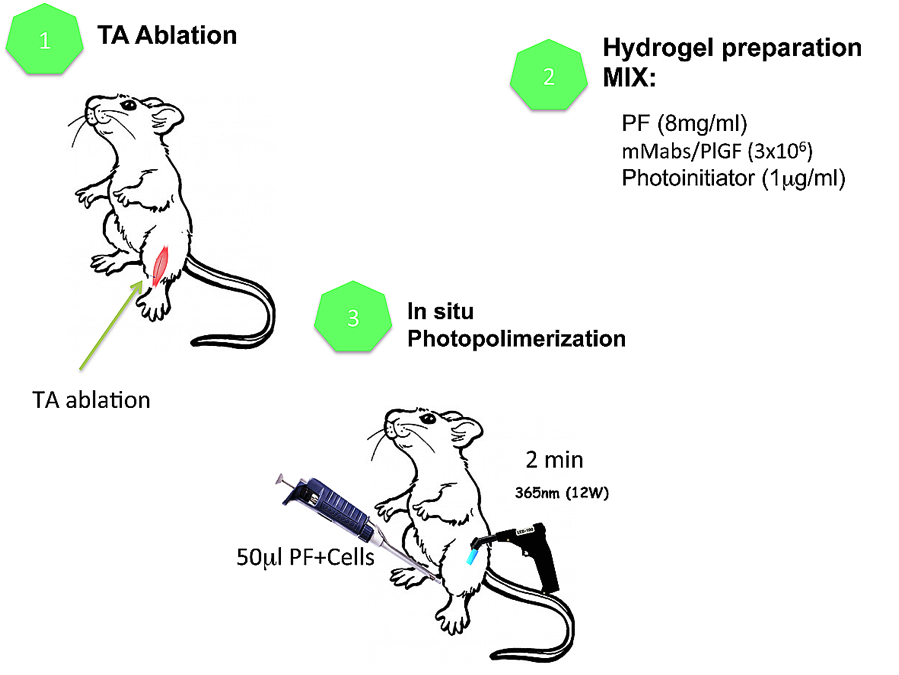


**Supplementary Fig. 13. Diagram depicting experimental procedures for ablated TA replacement.**


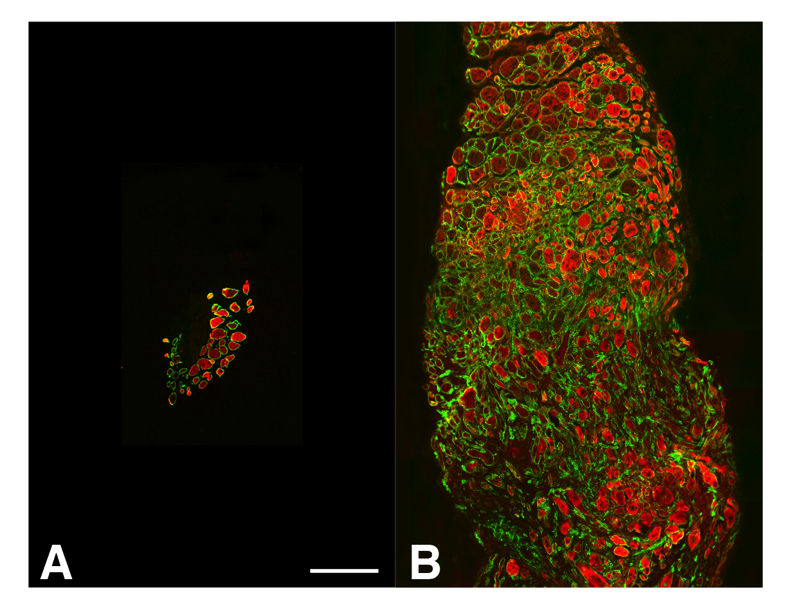


**Supplementary Fig. 14**.  **Immunoflorescence on cross section from acellular PF and PF embedded Mabs grafted in an ablated TA injury, revealing artifical muscle formation after 8weeks. (A)** Acellular PF implant into ablated TA venue labeled by MyHC (red) and laminin (green) revealing poor muscle myofibers at 8 weeks after TA massive ablation. **(B)** PF embedded Mabs graft replacing ablated TA at 8 weeks after TA removal revealing muscle reformation represented by several MyHC positive (red) myofibers surrounded by laminin (green) at 8 weeks from TA massive ablation. Scale bar value: (A, B) 100μm.

**Supplementary Tab. 1** Table resuming number of muscle satellite cells clone LacZ negative and/or positive scored in three different muscles.

| **Muscle** | **N. LacZ+ Clones** | **N. LacZ- clones** |
| --- | --- | --- |
| **Tibialis Anterior** | **0** | **117** |
| **Artificial** | **93** | **11** |

**Supplementary Tab. 2.** Table summarizing *in vivo* experimental animal groups were not indicated experiment are performed by mMabs.

| Group 1:  **Subcutaneous.**  **Back skin**  Rag2γchian-/-  mice  n=5 | Group 2:  **Subcutaneous.**  **TA**  Rag2γchian-/- mice  n=5 | Group 3:  **Subcutaneous.**  **TA (hMabs)**  SCID mice  n=5 | Group 4:  **TA ablation**  a.TA ABL-CONT  b.TA ABL-PF CONT  c.TA ABL-PF+MABS  SCID mice  n=10 | Group 5:  **Atrophy**  (denervation)  Rag2γchian-/- mice  n=4 | Group 6:  **Hypertrophy**  (Clenbuterol administration  2mg/Kg)  Rag2γchian-/-  mice  n=4 |
| --- | --- | --- | --- | --- | --- |
| 15 days | 4 weeks | 30 days | 10 days | 1 month | 1 month |
| 30 days | 8 weeks |  | 6 Months |  |  |
