# Supplementary figures and images for: In vivo generation of a mature and functional artificial skeletal muscle
Source: EMBO Mol Med. 2015 Feb 25;7(4):411–22. doi: 10.15252/emmm.201404062 (PMC4403043; doi:10.15252/emmm.201404062)

Original plate for Fig. 1 western blot

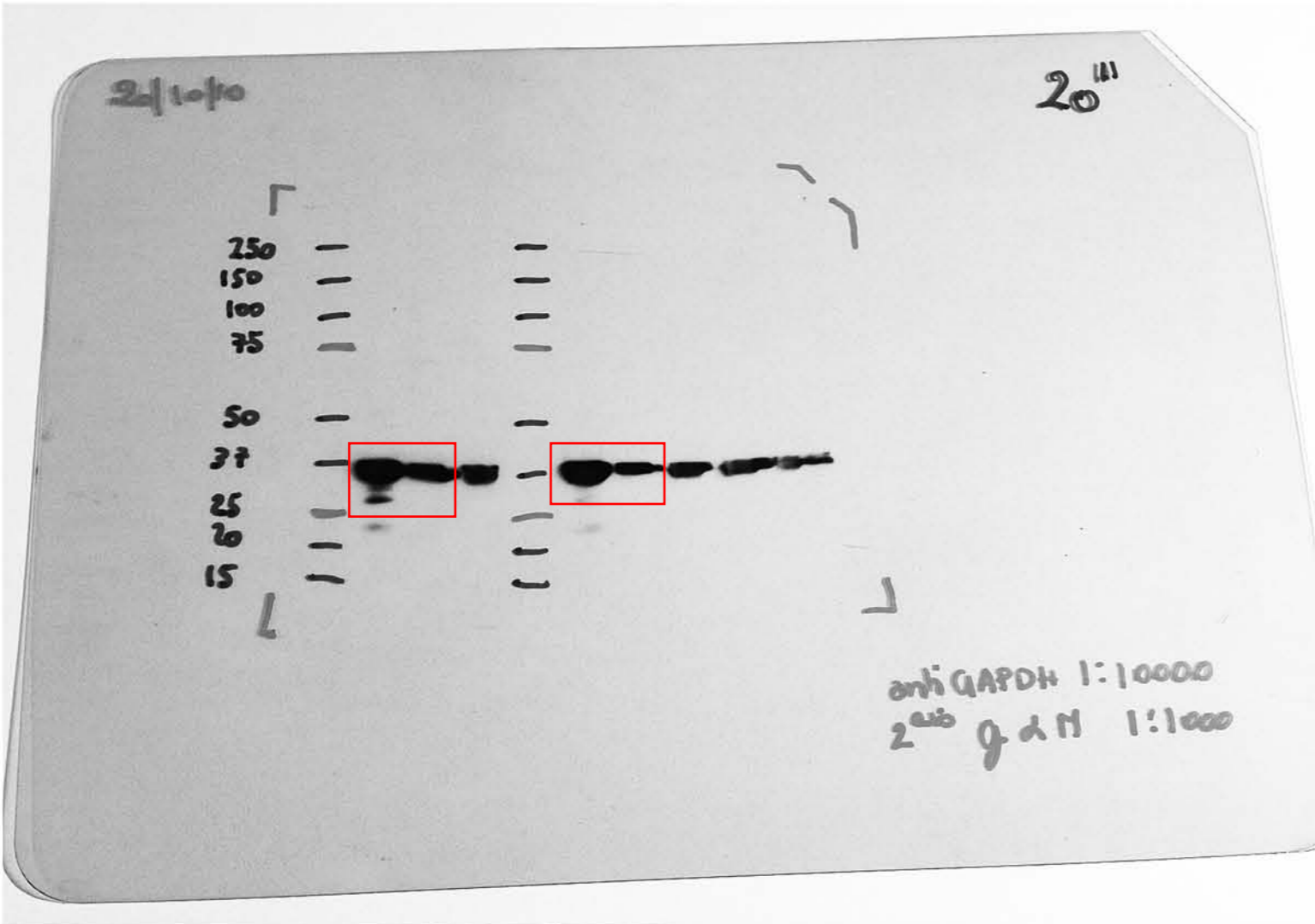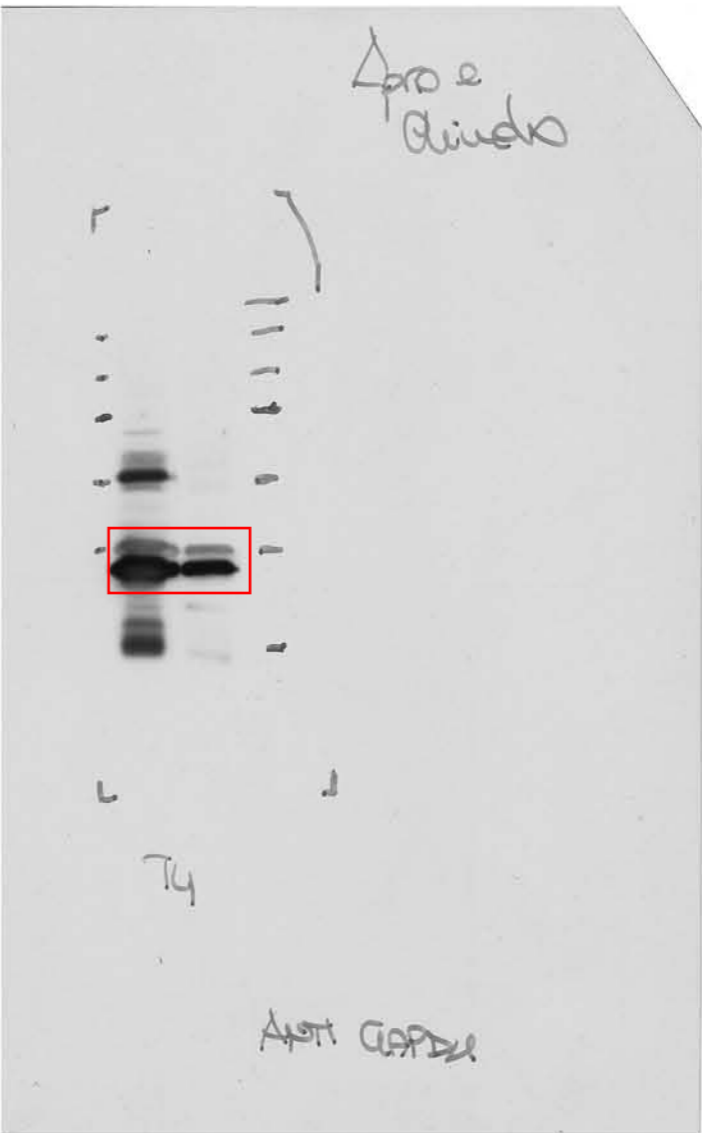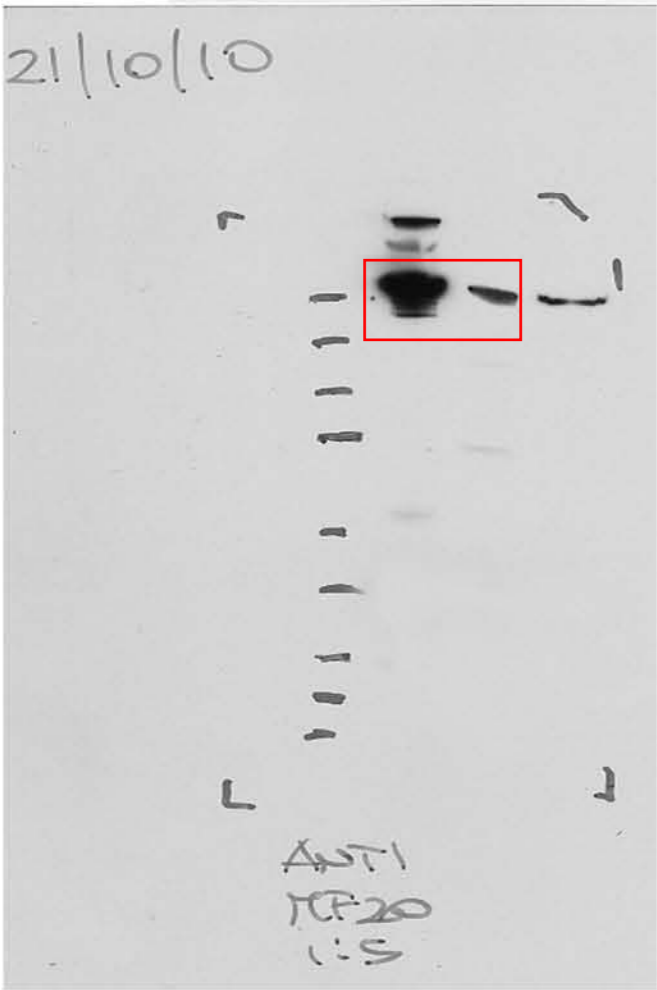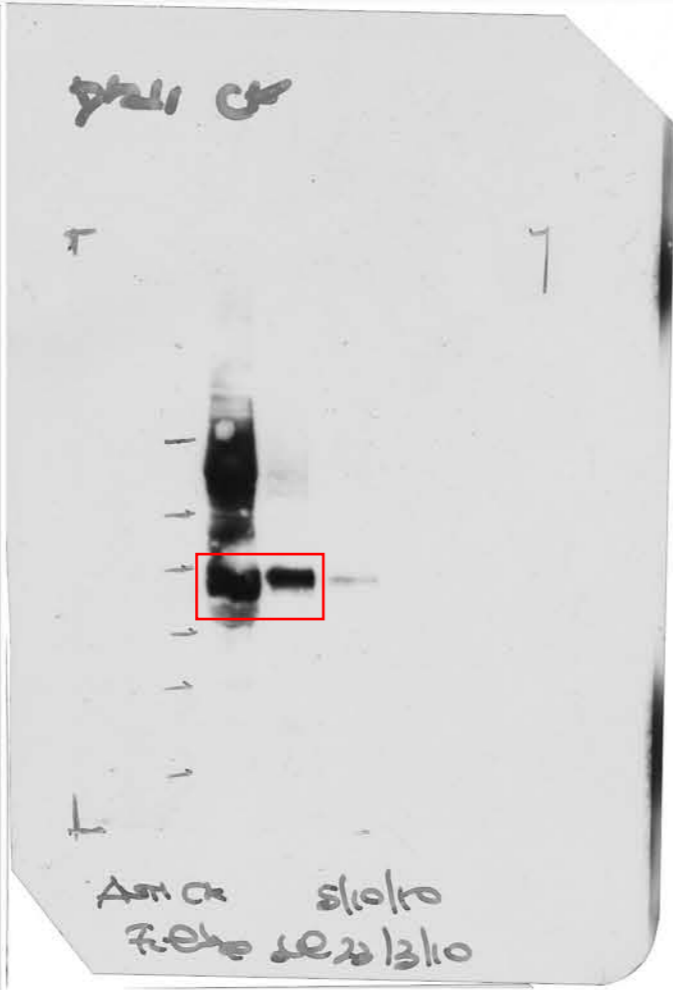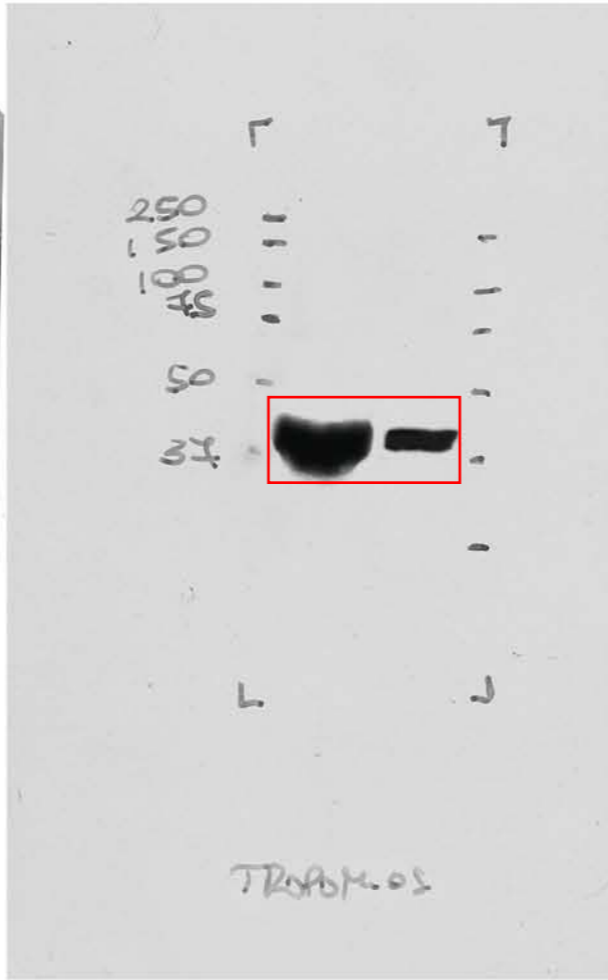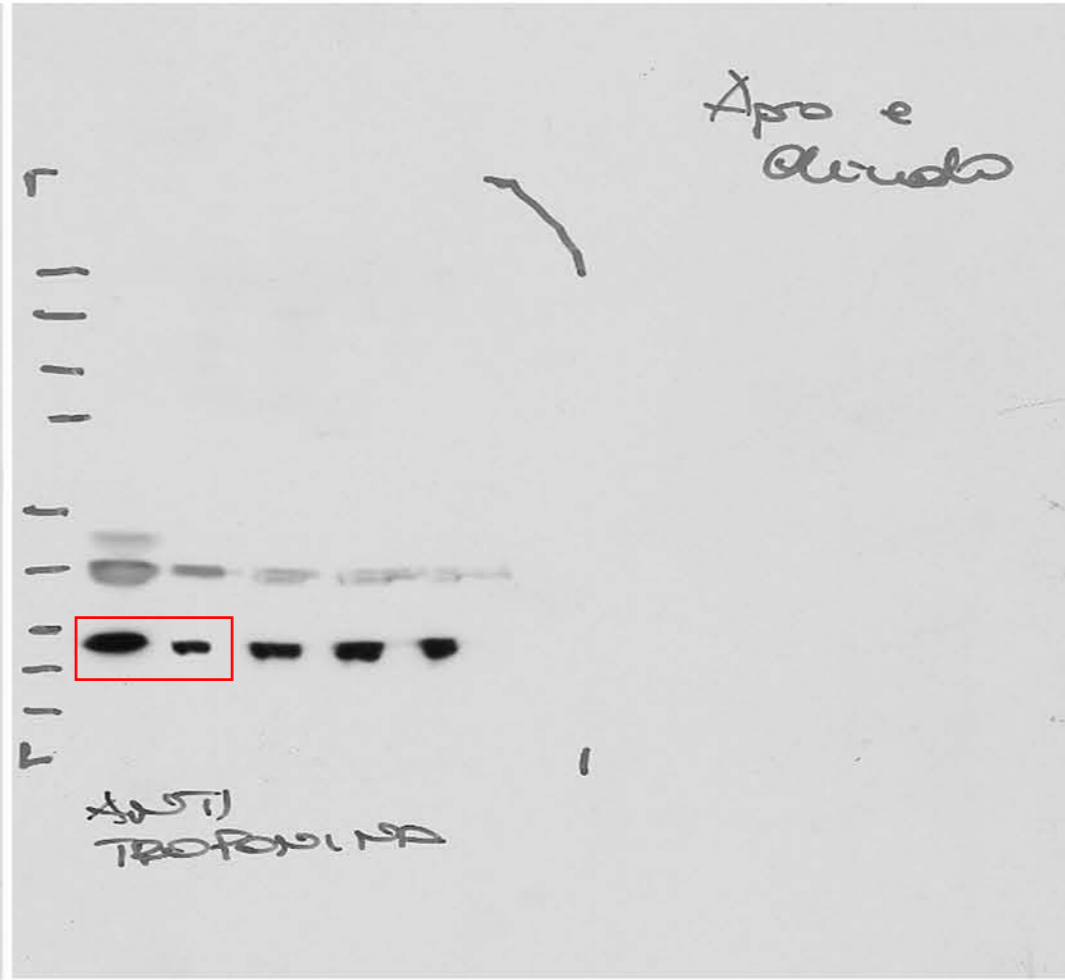

Supplement: Supplementary file 7 — Source Data for Figure 1 [file emmm0007-0411-sd7.pdf]

Source Data Figure 4W-Y

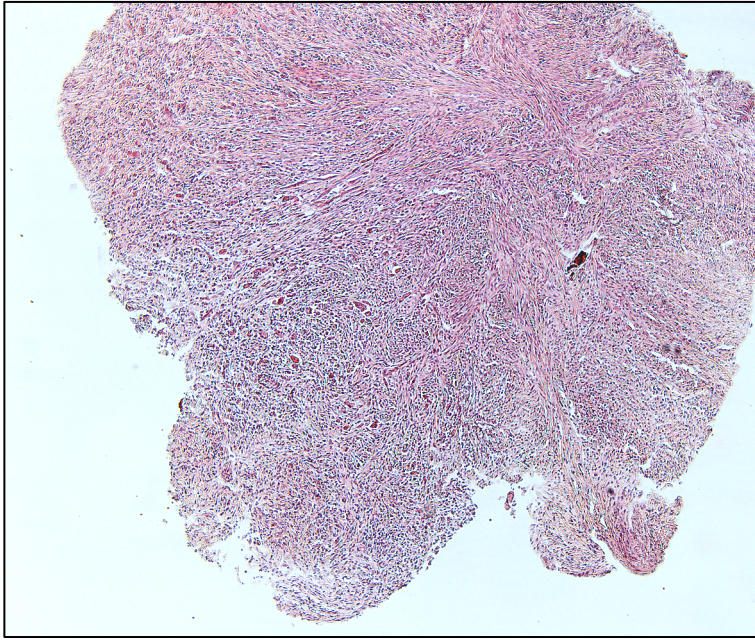

PF+Mabs 10gg 5Xa

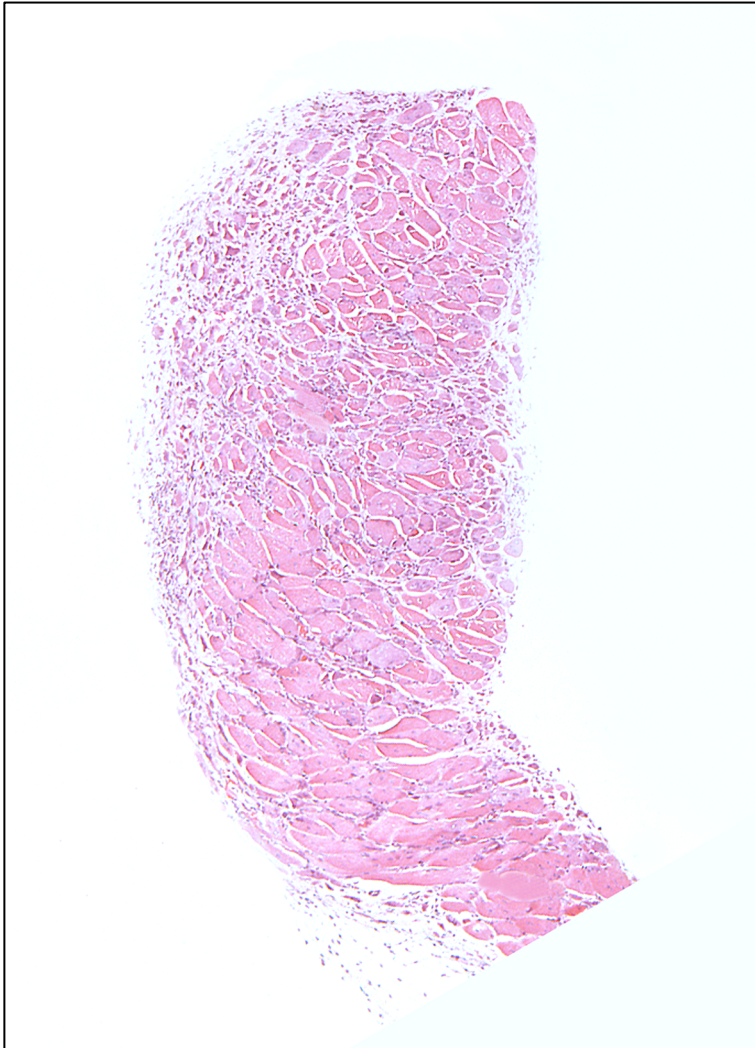

PF+Mabs 6 months 5Xa

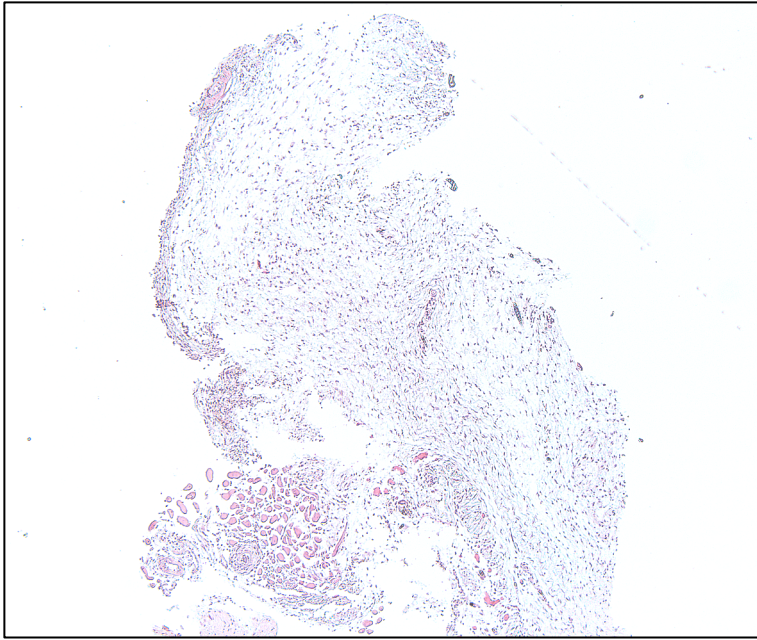

Control 10gg 5Xa

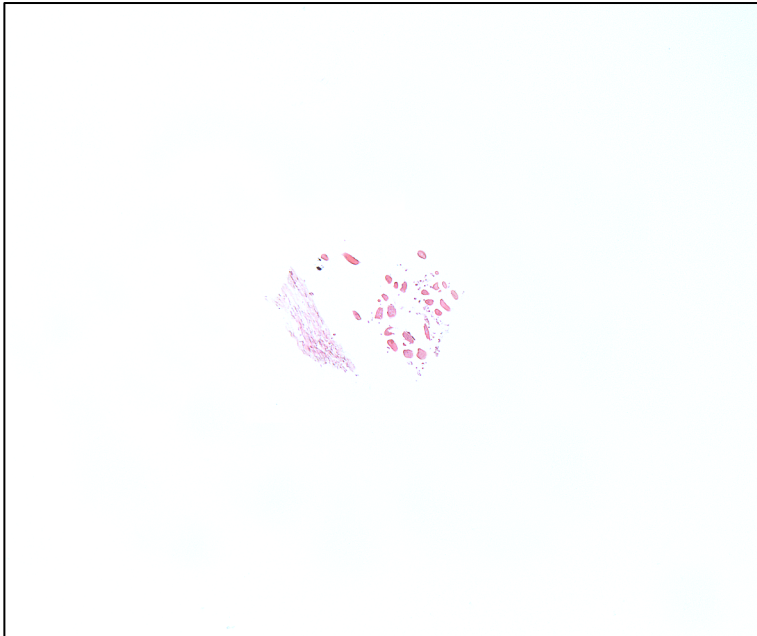

Control 6 months 5Xa

Supplement: Supplementary file 9 — Source Data for Figure 4 [file emmm0007-0411-sd9.pdf]
