# Supplementary material for: In vivo generation of a mature and functional artificial skeletal muscle
Source: EMBO Mol Med. 2015 Feb 25;7(4):411–22. doi: 10.15252/emmm.201404062 (PMC4403043; doi:10.15252/emmm.201404062)

Original plate for Fig. 2 western blot

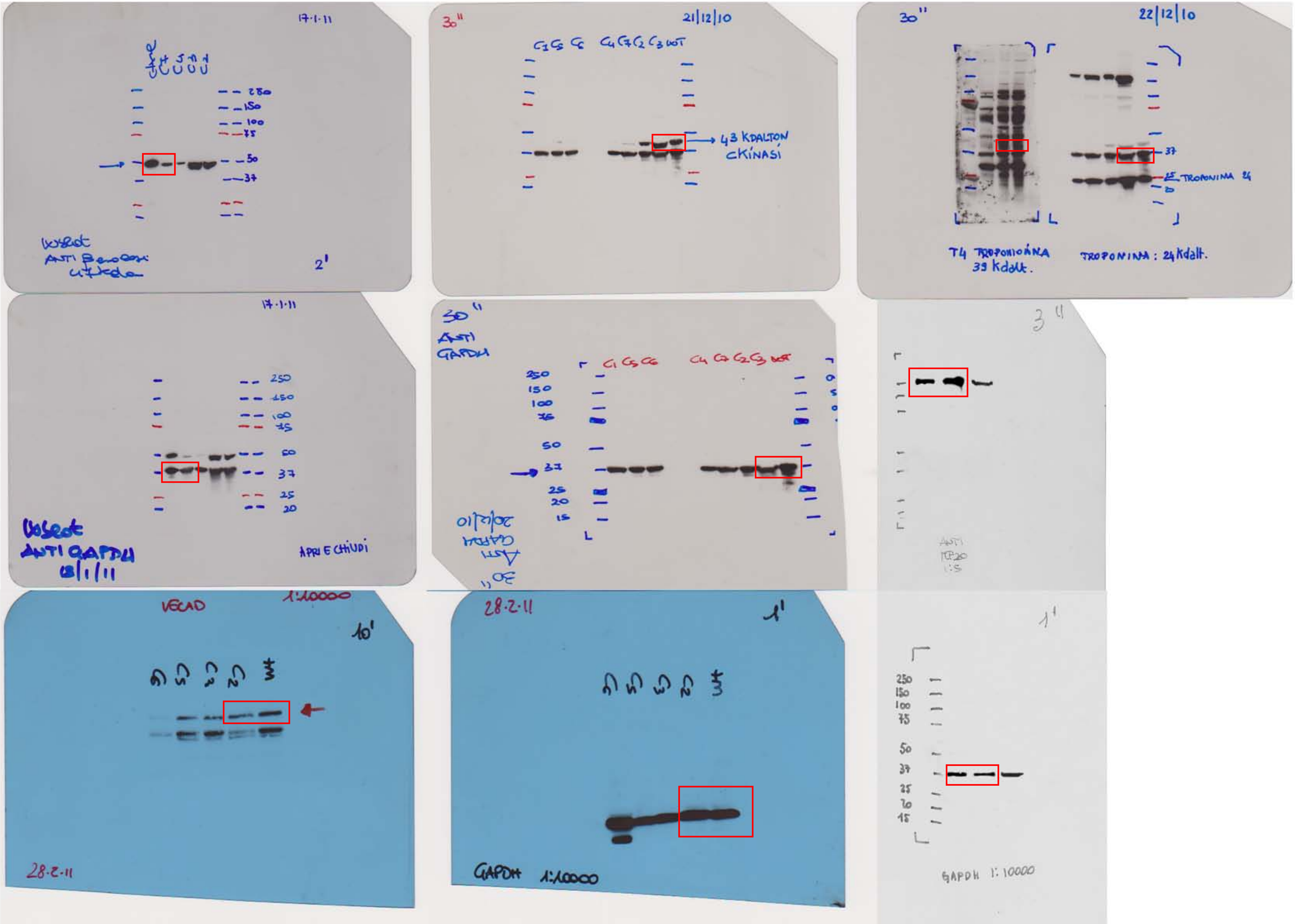

Supplement: Supplementary file 8 — Source Data for Figure 2 [file emmm0007-0411-sd8.pdf]
